# Correction to: BANMF-S: a blockwise accelerated non-negative matrix factorization framework with structural network constraints for single cell imputation

**DOI:** 10.1093/bib/bbaf034

**Published:** 2025-01-16

This is a correction to: Jiaying Zhao, Wai-Ki Ching, Chi-Wing Wong, Xiaoqing Cheng, BANMF-S: a blockwise accelerated non-negative matrix factorization framework with structural network constraints for single cell imputation, *Briefings in Bioinformatics*, Volume 25, Issue 5, September 2024, https://doi.org/10.1093/bib/bbae432

In equation 6, the position of hyperparameters γ_1_ and γ_2_ have been corrected from those in the originally-published paper. This change does not affect the numerical results or stated conclusions.

